# Insulator Defect Detection Algorithm Based on Improved YOLOv11n

**DOI:** 10.3390/s25051327

**Published:** 2025-02-21

**Authors:** Junmei Zhao, Shangxiao Miao, Rui Kang, Longkun Cao, Liping Zhang, Yifeng Ren

**Affiliations:** The College of Electrical and Control Engineering, North University of China, Taiyuan 030051, China

**Keywords:** insulator defect detection, you only look once (YOLO), multidimensional dynamic convolutions (ODConv)

## Abstract

Ensuring the reliability and safety of electrical power systems requires the efficient detection of defects in high-voltage transmission line insulators, which play a critical role in electrical isolation and mechanical support. Environmental factors often lead to insulator defects, highlighting the need for accurate detection methods. This paper proposes an enhanced defect detection approach based on a lightweight neural network derived from the YOLOv11n architecture. Key innovations include a redesigned C3k2 module that incorporates multidimensional dynamic convolutions (ODConv) for improved feature extraction, the introduction of Slimneck to reduce model complexity and computational cost, and the application of the WIoU loss function to optimize anchor box handling and to accelerate convergence. Experimental results demonstrate that the proposed method outperforms existing models like YOLOv8 and YOLOv10 in precision, recall, and mean average precision (mAP), while maintaining low computational complexity. This approach provides a promising solution for real-time, high-accuracy insulator defect detection, enhancing the safety and reliability of power transmission systems.

## 1. Introduction

The reliability and safety of electrical power systems are critical [1,2], with transmission line insulators playing a vital role in supporting and securing high-voltage lines [3,4]. Insulators provide essential electrical isolation and mechanical support, preventing the current from flowing between the conductor and the supporting structure [5,6]. However, prolonged exposure to environmental factors such as wind, rain, dust, and pollution makes insulators susceptible to issues like self-explosion, breakage, and dirt flash [7,8]. These problems pose significant risks to the safe operation of the transmission system, highlighting the need for effective inspection and maintenance strategies.

Various inspection techniques have been proposed for detecting insulator defects, including visual inspection, acoustic wave detection, infrared thermal imaging, and non-contact radar ranging [9,10,11]. Traditionally, manual inspections using handheld detection tools have been employed. However, the increasing use of drones in power systems has enabled aerial imagery for monitoring insulators along high-voltage transmission lines. Drones equipped with image processing algorithms facilitate more efficient inspections, but challenges remain [12]. The wide-angle perspective and varying viewpoints of drone imagery can result in images with small targets, complex backgrounds, and reduced detection accuracy. This leads to weak feature map representation and difficulty in extracting key information.

In recent years, deep learning models for object detection have gained considerable attention [13,14]. Learning paradigms can be categorized into four types: supervised learning, semi-supervised learning, weakly supervised learning, and self-supervised learning [15]. The classification depends on how the label information from the training data is utilized. Supervised learning relies on large amounts of labeled data for training. This enables the model to clearly learn the mapping between inputs and outputs. It is highly interpretable and relatively easy to implement. Semi-supervised learning uses a small amount of labeled data combined with a large volume of unlabeled data. This approach effectively reduces labeling costs. However, it comes with a higher algorithmic complexity and is sensitive to the quality of the unlabeled data [16]. Weakly supervised learning involves weaker label information. As a result, the model may struggle to fully capture all features and patterns in the data. Consequently, its performance is often inferior to that of supervised learning models, which rely on precise labels [17]. Self-supervised learning does not depend on manually labeled data. It generally exhibits stronger generalization capabilities, especially on large-scale datasets [18]. However, this method requires a deep understanding of both the data characteristics and the specific task. Additionally, the task design is complex, and achieving optimal results is not always guaranteed.

The YOLO algorithm, a widely used supervised learning-based object detection method, has proven effective in this context. Researchers have introduced various improvements to enhance its performance in diverse application scenarios. These enhancements include increasing the network’s depth or width to extract richer features, fusing multi-scale features for more accurate detection, and optimizing the loss function. Notable advancements include Zhang’s integration of the Ghost module into the YOLOv5 backbone and neck, reducing model size while improving performance on UAV platforms [19]. Han further refined YOLOv4 by incorporating the lightweight GhostNet module, which uses depthwise separable convolutions in the feature fusion layer [20]. Zheng achieves a 3.7% improvement over the YOLOv7 baseline model by implementing K-means++ anchor box optimization and a joint training strategy integrating the SIoU (SCYLLA-IoU) loss function with focal loss [21]. Additionally, Yu replaced the CIOU loss function with poly loss to address class imbalance, particularly for small targets, thereby improving detection accuracy by adjusting parameters based on different datasets [22]. Other significant developments include the DSMH-YOLOv4 [23], CACS-YOLO [24], SE-YOLOv5 [25], YOLOv5s-KE [26], and IL-YOLO algorithms [27]. Although these innovations have enhanced detection performance, further improvements are still needed.

To address these challenges and enhance detection accuracy, we propose a lightweight neural network based on the YOLOv11n architecture. The key contributions of our approach are as follows:

- The C3k2 module within the backbone network was redesigned based on ODConv, leading to the proposition of the C3k2_ODConv module, in which the two ordinary convolutions in the original bottleneck structure are replaced by multidimensional dynamic convolutions, thereby effectively enhancing the feature extraction capability for irregular defects.
- Slimneck replaces the neck component of YOLOv11n, reducing both the model’s parameter count and computational complexity.
- The WIoU loss function is introduced to optimize the anchor frames to more accurately locate defect positions and speed up network convergence.

The remainder of this paper is structured as follows: In Section 2, the YOLOv11 algorithm and the proposed methodology are discussed. Subsequently, Section 3 presents the results obtained from the application of the proposed method. Finally, Section 4 concludes with a comprehensive summary of the findings.

## 2. Methods

### 2.1. YOLOv11 Algorithm

The YOLOv11 model builds upon the CSP network and enhances the C2F module from YOLOv8 by introducing the C3K2 module. The new C3K2 module serves as the core structure for both feature extraction and fusion, significantly enhancing the model’s ability to capture key features and identify detailed target characteristics. Compared to YOLOv10, YOLOv11 improves the PSA module (after the SPPF module) by upgrading it to the C2PSA module.The C2PSA network extends the C2F module by integrating positional self-attention, which is achieved through stacking multiple PSA modules, splitting the input tensor, and combining the outputs via a final convolution layer. This architecture enables the model to capture more complex features by mapping the input to a higher-dimensional space, which facilitates the learning of intricate linear relationships and richer feature representations.Additionally, YOLOv11 incorporates the HEAD concept from previous YOLO models and applies depth-separable convolution to the detection head’s branching structure. This modification reduces redundant computations, enhancing operational efficiency. A residual structure is selectively introduced to optimize gradient propagation, further improving the model’s training effectiveness. Furthermore, YOLOv11’s feature extraction capabilities are enhanced through the integration of attention mechanisms and feed-forward neural networks, boosting overall performance [28,29].

### 2.2. Proposed Method

#### 2.2.1. Improved C3k2 Module

**Figure 1:**
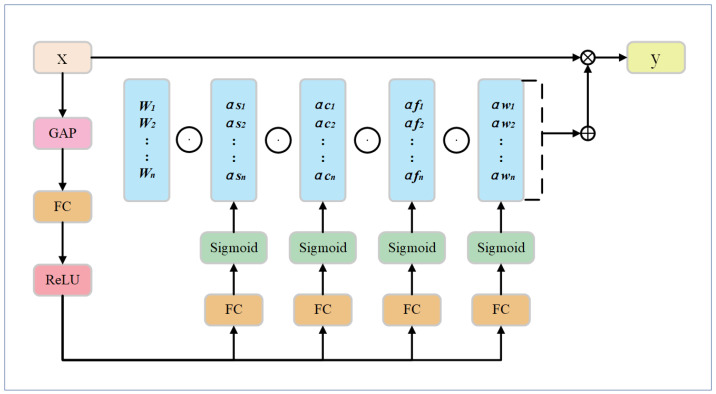
ODConv Structure.

In this paper, the C3k2 module in the backbone network was redesigned based on ODConv. Different from the C3k2 module, the C3k2_ODConv module replaces the two ordinary convolutions of the original bottleneck with a multidimensional dynamic convolution. C3k2_ODConv can effectively improve the feature extraction ability of irregular defects, as well as improve the performance of the detection of defects such as cracks. The structure of the ODConv module is shown in Figure 1.

ODConv introduces an innovative convolution method that enhances the performance of convolutional neural networks (CNNs) by integrating attention mechanisms across four distinct dimensions. In the spatial location dimension, $\alpha_{si}$ assigns attention scalars to each parameter of the convolution kernel at k×k spatial positions, enabling the kernel to dynamically focus on different input locations. In the input channel dimension, $\alpha_{ci}$ applies weights to each input channel of the convolution filter, emphasizing relevant channel information. Similarly, in the output channel dimension, $\alpha_{fi}$ allocates attention scalars to different convolution filters, refining feature specificity. Lastly, $\alpha_{wi}$ adjusts the overall intensity of the convolution kernel, optimizing its output. These four attention mechanisms work synergistically, dynamically adjusting the convolution operation at multiple levels based on the input, thereby significantly improving the CNN’s feature extraction capability [30]. Notably, ODConv matches or outperforms traditional methods like CondConv and DyConv while introducing far fewer additional parameters. The calculation formula is shown below:

(1) $$y=\left(\sum_{i=1}^{n}\alpha_{wi}⊙\alpha_{fi}⊙\alpha_{ci}⊙\alpha_{si}⊙W_{i}\right)*x,$$

where $W_{i}$ is expressed as the *i* th convolutional kernel, *y* is indicated as the output feature vector, and *x* is indicated as the input feature vector.

**Figure 2:**
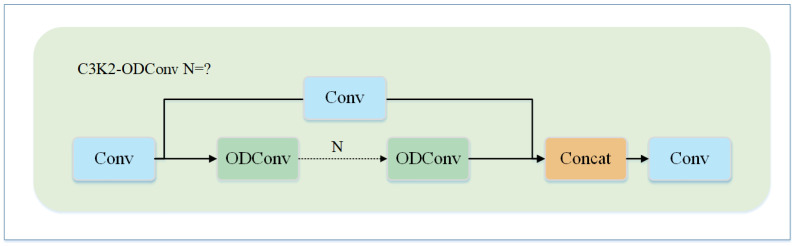
C3k2-Conv Structure.

I-type and V-type strings are commonly used in power lines as overhanging string insulators, and their structural characteristics dictate that they necessarily overlap each other and are covered in the image [31]. For irregular and large-area defects such as cracks, it is not enough to only rely on local features. In order to detect such defects more accurately, the model needs to focus on more multi-dimensional global information. Therefore, the use of C3k2_ODConv in the backbone network can extract more information and thus improve the detection accuracy of defects such as cracks.The structure of the C3k2_ODConv module is shown in Figure 2.

#### 2.2.2. Slimneck

Slimneck introduces an efficient neck solution (SNs) for real-time detection architectures, improving both performance and computational efficiency. This is achieved through innovative designs that enhance accuracy while minimizing computational costs. A key feature of Slimneck is the GSConv method, which addresses the limitations of depthwise separable convolution (DSC). While DSC reduces parameters and computational load, it struggles with channel information separation, limiting feature representation. GSConv combines the benefits of standard convolution (SC) and DSC through using a shuffling mechanism to integrate features from both, preserving channel connections. This hybrid approach approximates SC’s feature representation power while reducing computational costs by 50%, capturing more spatial and channel features and significantly improving accuracy [32].

Specifically, the GSConv in the Slimneck module divides the input features into multiple groups. It then performs independent convolution operations on each group, which reduces the number of parameters associated with the convolution. Instead of applying a full convolution to all of the channels of the input features, the GSConv processes each group separately. This significantly reduces both the computational cost and the number of parameters involved. Additionally, the GSConv employs the channel shuffle operation. After depthwise separable convolution, the channels are shuffled to enable interactions between them. This helps the output of the depthwise separable convolution approximate the result of a standard convolution, improving the ability to extract and fuse features without incurring excessive computational costs.

Another important component is that the VoV-GSCSP module, which is built upon the GSConv bottleneck, utilizes a one-shot aggregation strategy. This module reduces computational complexity and inference time while maintaining accuracy. The VoV-GSCSP1 configuration is simple and efficient, while VoV-GSCSP2 and VoV-GSCSP3 prioritize increased feature reuse. Due to hardware compatibility, VoV-GSCSP1 is more widely adopted in practical applications. Additionally, integrating feature enhancement techniques such as visual attention mechanisms further improves detection performance. For example, spatial pyramid pooling (SPP), which is placed at the end of the backbone network, employs max pooling at multiple scales to capture a larger receptive field and to recalibrate spatial feature weights. This allows the model to focus on important features, thereby enhancing detection accuracy [33].

#### 2.2.3. WIoU Loss Function

The WIoU loss function incorporates a dynamic non-monotonic focusing mechanism, which is designed based on a detailed analysis of anchor box characteristics and target processing needs.This mechanism modulates the attention assigned to different anchor boxes, enabling the model to focus more selectively on anchor boxes that align with the specific features of the targets. From a gradient optimization perspective, this mechanism helps mitigate the interference of deleterious gradients during model training, improving convergence accuracy. Moreover, by rationally distributing attention among anchor boxes, the model’s generalization ability across diverse data distributions is enhanced [34,35].

The WIoU loss function optimizes anchor box processing through a dynamic, non-monotonic focusing mechanism. This mechanism evaluates the quality of anchor boxes and assigns differentiated gradient values. A low outlier degree signifies a high overlap between the anchor box and the ground-truth box, indicating high quality. In this case, a reduced gradient gain is applied to avoid over-optimization. In contrast, a high outlier degree indicates low overlap, reflecting low quality, and it results in a smaller gradient gain that helps to mitigate harmful gradients. A medium outlier degree suggests an intermediate level of quality, leading to a larger gradient gain and making the anchor box a primary target for model optimization. By dynamically classifying the quality of anchor boxes, WIoU avoids the excessive penalization of low-quality anchor boxes, which is a common issue in traditional IoU-based methods. At the same time, it reduces gradient competition among high-quality anchor boxes.

The WIoU loss function is calculated is as follows:

(2) $$L_{WIoU}=rR_{WIoU}L_{IoU},$$

(3) $$r=\frac{\beta }{\delta \alpha^{\beta -\delta }},$$

(4) $$\beta =\frac{L_{IoU}^{*}}{L_{IoU}}\in \left[0,+\infty \right),$$

(5) $$R_{WIoU}=exp\left(\frac{{\left(x-x_{gt}\right)}^{2}+{\left(y-y_{gt}\right)}^{2}}{(W_{g}^{2}+H_{g}^{2})*}\right),$$

(6) $$L_{IoU}=1-IoU.$$

Here, $L_{WIoU}$ represents the WIoU loss function, and *r* denotes the non-monotonic focusing factor. The term $R_{WIoU}$ refers to the penalty term, while $L_{IoU}$ represents the loss function. Additionally, β indicates an outlier, δ corresponds to gradient gain, and α is the balance factor. The term $L_{IoU}^{*}$ denotes the monotonic focusing factor. The coordinates of the center point of the anchor frame are represented by (x,y), while $\left(x_{gt},y_{gt}\right)$ refers to the coordinates of the target box center. Finally, $W_{g}$ and $H_{g}$ represent the width and height of the bounding boxes for the anchor and target, respectively.

**Figure 3:**
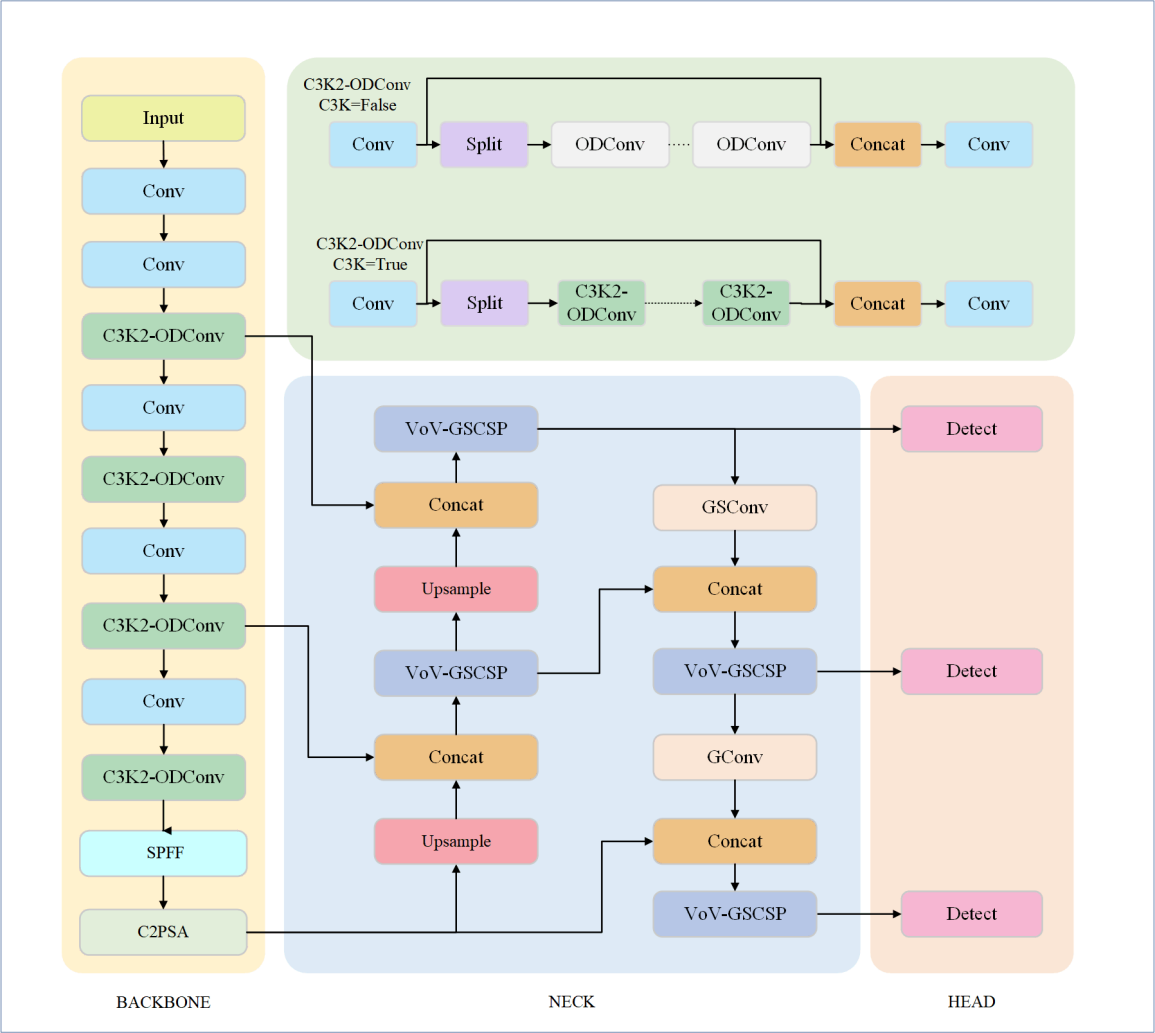
The improved YOLO11n Model Structure.

The improved YOLO11n network model is shown in Figure 3. In terms of structural design, the backbone employs ODConv, which replaces the standard convolution in the traditional C3K2 module. This replacement enhances feature extraction by leveraging four dimensions: spatial position, input channels, output channels, and convolution kernel strength. In the next section, the Slimneck structure is introduced to fuse the feature information generated by standard convolutions with the output feature maps from depth convolutions. This fusion makes the output feature maps from depth convolutions more similar to those from standard convolutions, effectively reducing computational complexity while preserving key channel information. Regarding the training strategy, the model incorporates the WIoU loss function, optimizing the anchor box selection process through a dynamic focusing mechanism.

## 3. Experiments and Results

### 3.1. Experimental Implementation

#### 3.1.1. Experiment Platform

The experiment was conducted using the Pytorch 1.10.0 deep learning framework, with Python 3.9 as the programming language. The system was operated on Windows 10 utilizing a 13th Gen Intel(R) Core(TM) i7-13700K 3.40 GHz CPU, which was paired with 64 GB of RAM. The GPU used for processing was an NVIDIA GeForce RTX4090 equipped with 24 GB of memory. Additional information on the model’s training phase is available in Table 1.

#### 3.1.2. Dataset

The experiment utilized a custom-built dataset comprising defective insulators on transmission lines. A total of 1628 original images were captured by a company affiliated with the state grid, with which the research team collaborated. These images were obtained using imaging devices mounted on drones. To enhance the data’s utility and ensure its reliability, data cleaning procedures were applied. Duplicates were removed, and images that were blurry, improperly exposed, or severely occluded were excluded from the dataset. For data annotation, the professional tool Labellmg was used. Annotation accuracy and consistency were ensured through a rigorous review process that was established by the research team. As a result, a refined dataset of 1558 images was created. The dataset was then divided into training, validation, and test sets in a ratio of 8:1:1. The insulators were classified into three categories: “insulator”, “broken”, and “pollution-flashover”. These categories contained 512, 528, and 518 images, respectively.

#### 3.1.3. Evaluation Indicators

Precision (*P*) quantifies the proportion of true positive samples among those predicted as positive. Its calculation formula is as follows:

(7) $$P=\frac{TP}{TP+FP}.$$

Here, TP (True Positives) denotes the number of samples correctly predicted as positive, while FP (False Positives) represents the number of samples incorrectly predicted as positive.

Recall (*R*) measures the proportion of actual positive samples correctly predicted by the model. It is calculated as follows:

(8) $$R=\frac{TP}{TP+FN}.$$

In this context, FN (False Negatives) refers to the number of true positives incorrectly predicted as negative.

Mean average precision (mAP) is a widely used evaluation metric in object detection that assesses model accuracy across various thresholds. This study employed two mAP metrics: mAP50 and mAP50-95.

- mAP50: The mean average precision at an intersection over union (IoU) threshold of 0.50.
- mAP50-95: The mean average precision calculated at IoU thresholds ranging from 0.50 to 0.95 (in increments of 0.05).

GFLOP (giga floating-point operations per second) denotes the number of floating-point operations executed per billion cycles per second. It is a standard metric used to assess the computational complexity of a model. A lower GFLOP value typically signifies greater computational efficiency on devices with constrained resources. Conversely, a higher GFLOP value often necessitates more advanced hardware to achieve real-time processing performance.

FPS (frames per second) denotes the rate at which an object detection algorithm processes frames. A higher FPS indicates that the algorithm can process a greater number of images within a given time interval.

### 3.2. Comparative Experiment

To evaluate the performance of the improved model in insulator defect detection, we conducted comparative experiments with classic object detection networks. The highly cited paper of [36] offers a comprehensive review of the structural advancements, advantages, and limitations of each iteration of the YOLO series algorithms, emphasizing their authority and widespread application in surface defect detection within industrial settings. Based on this review, the YOLO series algorithms were selected as the benchmark for this study, thereby facilitating the comparison of our results against a widely recognized standard. In addition, other models, such as Fast-RCNN and SSD, were incorporated as supplementary benchmarks. Except for the improved model proposed in this paper, all of the models used in the comparative experiments were official models. The experiments were conducted on the validation set of a self-built dataset to ensure that the model accurately meets the specific task requirements set by our partner company, the results of which are presented in Table 2.

**Figure 4:**
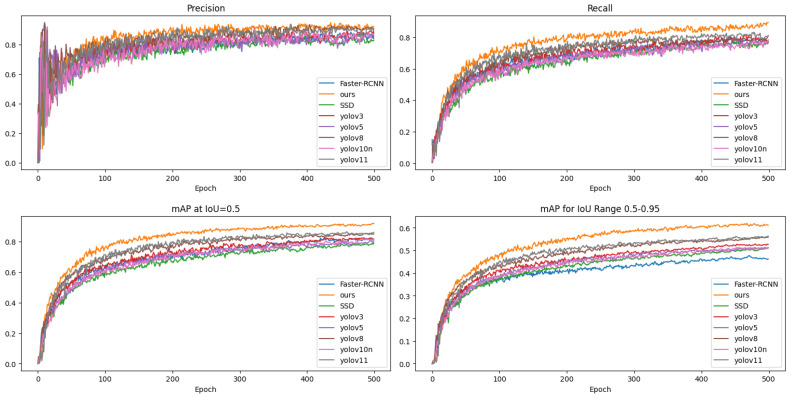
Performance comparison of the different models.

In addition, to intuitively display the results, this study plotted the comparison charts of precision, recall, mAP50, and mAP50-95 (Figure 4), which helped us to gain an insight into the performance differences of the various models under different indicators and provided data support for subsequent research.

It can be clearly concluded from the experimental results and data that the improved YOLO11 model significantly outperformed the original YOLOv11, YOLOV10, and other models in terms of performance. Compared with the mean average precision of 87.3% of the original YOLOv11, the average precision of the improved YOLO11 model increased by 3.7%. When compared with the average precision of 83.8% of YOLOV10, the improvement rate reached 7.2%. Moreover, while achieving a substantial increase in detection speed, the improved YOLO11 model had also been effectively optimized in terms of the number of parameters and computational complexity, maintaining them at a relatively low level. Based on maintaining a high accuracy rate, this model exhibited a relatively high recall rate. In terms of both accuracy and recall, all of the indicators were superior to those of other comparative models, demonstrating obvious comprehensive performance advantages in the insulator defect detection task.

Different loss functions such as EIoU, DIoU, SIoU, and GIoU were selected to compare with the original loss function CIoU of YOLOv11 and the WIoU selected in this paper, where the aim was at verifying the effects of different loss functions on network performance. The experimental results, taking YOLOv11n as the benchmark model, are shown in Table 3, where Y-xIoU and so on indicate that different loss functions were introduced into the benchmark model. The experimental results show that, compared with the original loss function of YOLOv11 and several other loss functions, the introduction of WIoU into the model was more effective in improving the network performance on the dataset. This may be due to the fact that WIoU focuses on weighting target objects of different sizes, whereas the insulator images collected by UAVs are themselves of different sizes due to factors such as the shooting angle.

### 3.3. Ablation Experiment

In order to explore the improvement effects of the C3k2_ODConv module, Slimneck, and the loss function proposed in this paper within the network, groups of ablation experiments were conducted. The performance indicators of adding each model separately and the final improved model were obtained for comparison. The experimental results are shown in Table 4. Here, “Y” indicates that the module was added to the network.

Model 1 refers to the baseline YOLOv11n algorithm. Groups 2, 3, and 4 represent variants of the YOLOv11n algorithm, each incorporating C3k2_ODConv, Slimneck, and WIoU, respectively. These modifications resulted in corresponding improvements in the mAP50 of 0.8%, 0.3%, and 1.7%. Notably, the integration of Slimneck led to an 18.6% reduction in the model parameters and a 48 FPS increase, underscoring the module’s effectiveness in reducing both model complexity and computational cost while simultaneously enhancing inference speed. Group 5 corresponds to the algorithm that was obtained by the simultaneous incorporation of C3k2_ODConv and Slimneck, yielding a 1.8% increase in mAP50. Group 6 integrated C3k2_ODConv and WIoU, resulting in a 2% improvement in mAP50. Group 7 combined Slimneck and WIoU, achieving a 2.2% increase in mAP50. Finally, Group 8 represents the proposed algorithm in this study, which demonstrated a 3.7% increase in mAP50, a 4.5% improvement in mAP50-95, a 15.5% reduction in model parameters, and a 42 FPS enhancement. This effectively validated the effectiveness of the C3k2_ODConv module designed in this paper in enhancing the detection accuracy of the model. It also fully demonstrates that the incorporation of Slimneck and WIoU into the YOLOv11n algorithm system plays a positive and effective role in enhancing the detection performance of the model.

### 3.4. Visualization Experiment

**Figure 5:**
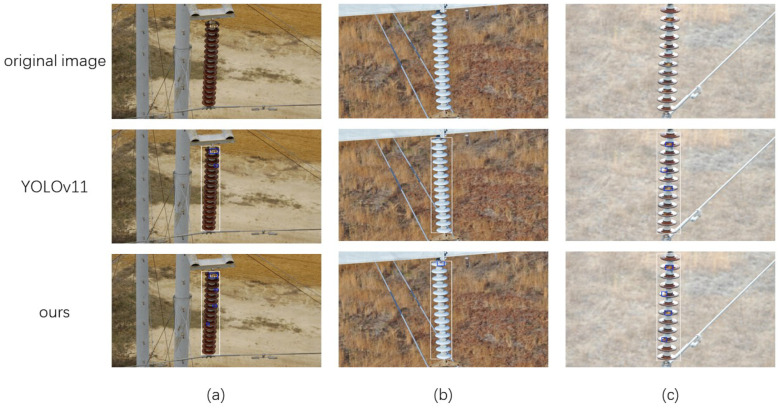
Comparison of the detection effects of the improved YOLOv11. (**a**) Visual comparison group 1; (**b**) Visual comparison group 2; (**c**) Visual comparison group 3.

Figure 5 presents the actual detection effects of the YOLOV11 algorithm before and after improvement. In the detection results of the pre-improvement YOLOV11 algorithm, the two cases of insulator pollution flashover in Figure (a) were not detected; one case of insulator pollution flashover near the end in Figure (b) failed to be recognized; and one small insulator pollution flashover defect in Figure (c) was also undetected. In sharp contrast, the improved YOLOv11 algorithm was able to accurately identify the flashover traces of the insulators.The above results fully demonstrate that, compared with the initial YOLOV11n algorithm, the improved algorithm proposed in this paper has more excellent detection performance and can more accurately identify the defective targets of insulators.

## 4. Conclusions

This study presents an improved YOLOv11n-based model for detecting defects in electrical transmission line insulators. The model incorporates several key innovations: the C3k2 module was redesigned with ODConv, Slimneck was introduced to reduce model size and computational load, and the WIoU loss function was applied for optimized anchor box processing. These enhancements significantly improve both detection accuracy and efficiency. Experimental results show that the enhanced model outperforms traditional approaches, including earlier YOLO versions and other object detection networks like Faster R-CNN and SSD. Specifically, the model achieved a precision of 91.8%, a recall of 88.1%, and an mAP50 of 0.91, demonstrating its effectiveness in detecting irregular defects in insulators. Comparative and ablation studies confirm the contributions of each module, with the combination of ODConv, Slimneck, and WIoU yielding superior performance. With higher detection accuracy and reduced computational complexity, this model is well suited for real-time insulator monitoring, providing a robust solution for ensuring the safety and reliability of power transmission systems. In future research, we aim to incorporate a broader range of defect types and data under more complex conditions to train and evaluate the model’s generalization performance. Furthermore, we plan to deploy the network on embedded devices for further investigation, with the goal of enhancing the detection capabilities of the YOLO algorithm for insulator defects.

## Figures and Tables

**Table 1:** Model hyperparameter settings.

Parameters	Setup
Batch size	64
Image size	640 × 640
Initial learning rate	0.01
Final learning rate	0.01
Weight decay	0.0005
Momentum	0.937
Optimizer	SGD

**Table 2:** Comparative experiments of the different models.

Model	P	R	mAP50	mAP50-95	GFLOPs
Faster-RCNN	0.856	0.783	0.817	0.477	50.6
SSD	0.83	0.774	0.798	0.514	24.2
YOLOv3	0.851	0.8	0.824	0.536	14.3
YOLOv5	0.885	0.802	0.836	0.537	13.8
YOLOv8	0.909	0.785	0.854	0.559	28.4
YOLOv10	0.85	0.806	0.838	0.541	14.3
YOLOv11	0.906	0.84	0.873	0.574	7.6
Ours	0.918	0.881	0.91	0.619	6.5

**Table 3:** Comparative experiments of the different loss functions.

Model	P	R	mAP50	mAP50-95
Y-CIoU	0.906	0.84	0.873	0.574
Y-EIoU	0.868	0.841	0.872	0.579
Y-DIoU	0.909	0.838	0.875	0.581
Y-SIoU	0.907	0.834	0.872	0.578
Y-GIoU	0.897	0.839	0.876	0.582
Y-WIoU	0.899	0.845	0.888	0.588

**Table 4:** Ablation experiment.

Model	C3k2_ODConv	Slimneck	WIoU	P	R	mAP50	mAP50-95	FPS	Params/M
Model 1				0.906	0.84	0.873	0.574	186	2.58
Model 2	Y			0.934	0.829	0.88	0.577	172	2.65
Model 3		Y		0.91	0.848	0.876	0.579	234	2.10
Model 4			Y	0.899	0.845	0.888	0.588	208	2.58
Model 5	Y	Y		0.908	0.858	0.889	0.594	214	2.32
Model 6	Y		Y	0.935	0.844	0.89	0.599	195	2.64
Model 7		Y	Y	0.912	0.843	0.893	0.593	241	2.15
ours	Y	Y	Y	0.918	0.881	0.91	0.619	228	2.18

## Data Availability

The datasets presented in this article are not readily available because they contain proprietary information protected under contractual agreements with the collaborating company. Requests to access the datasets should be directed to the corresponding author.
